# The Digital Fish Library: Using MRI to Digitize, Database, and Document the Morphological Diversity of Fish

**DOI:** 10.1371/journal.pone.0034499

**Published:** 2012-04-06

**Authors:** Rachel M. Berquist, Kristen M. Gledhill, Matthew W. Peterson, Allyson H. Doan, Gregory T. Baxter, Kara E. Yopak, Ning Kang, H. J. Walker, Philip A. Hastings, Lawrence R. Frank

**Affiliations:** 1 Center for Scientific Computation in Imaging, University of California San Diego, La Jolla, California, United States of America; 2 Marine Vertebrate Collection and Marine Biology Research Division, Scripps Institution of Oceanography, University of California San Diego, La Jolla, California, United States of America; 3 Center for Functional Magnetic Resonance Imaging, University of California San Diego, La Jolla, California, United States of America; University of Lethbridge, Canada

## Abstract

Museum fish collections possess a wealth of anatomical and morphological data that are essential for documenting and understanding biodiversity. Obtaining access to specimens for research, however, is not always practical and frequently conflicts with the need to maintain the physical integrity of specimens and the collection as a whole. Non-invasive three-dimensional (3D) digital imaging therefore serves a critical role in facilitating the digitization of these specimens for anatomical and morphological analysis as well as facilitating an efficient method for online storage and sharing of this imaging data. Here we describe the development of the Digital Fish Library (DFL, http://www.digitalfishlibrary.org), an online digital archive of high-resolution, high-contrast, magnetic resonance imaging (MRI) scans of the soft tissue anatomy of an array of fishes preserved in the Marine Vertebrate Collection of Scripps Institution of Oceanography. We have imaged and uploaded MRI data for over 300 marine and freshwater species, developed a data archival and retrieval system with a web-based image analysis and visualization tool, and integrated these into the public DFL website to disseminate data and associated metadata freely over the web. We show that MRI is a rapid and powerful method for accurately depicting the in-situ soft-tissue anatomy of preserved fishes in sufficient detail for large-scale comparative digital morphology. However these 3D volumetric data require a sophisticated computational and archival infrastructure in order to be broadly accessible to researchers and educators.

## Introduction

Natural history collections possess a wealth of anatomical, morphological, genetic and geographic data that are essential for documenting and understanding biodiversity [1]–[3]. Although some of the materials housed in fish collections include dried hard tissue preparations, cleared-and-stained specimens, and tissues samples for molecular genetic analysis, the bulk of these collections typically contain intact ‘wet’ specimens that have been preserved in formalin and post-fixed in alcohol for long-term storage. However, broad access to this material is commonly impeded by stringent storage requirements, which involve hazardous liquids, fragile storage containers (glass), and cramped storage facilities. Access is often further limited by the desire of curators to maintain the long-term integrity of specimens, especially in the case of holotypes and species that are delicate, rare, or difficult to obtain. This can conflict with specimen usage, which often requires the dissection, and sometimes even the destruction, of specimens in order to reveal internal anatomical and morphological detail.

As the cost of maintaining physical collections becomes increasingly expensive, the biology community is faced with the difficult problem of how to preserve these valuable research and education resources. One approach to this problem is to leverage the significant recent advances in imaging, software, and computer hardware technologies to develop digital libraries [4]–[7]. In principal, a comprehensive digital library would provide a complete set of tools and methods to collect, visualize, analyze, archive, and disseminate high resolution imaging data over the web. In practice, developing a comprehensive program that integrates all of these aspects is a significant technological and organizational task. In this paper we describe our current progress on the Digital Fish Library project (DFL, http://www.digitalfishlibrary.org), which aims to develop such a program with a particular focus on magnetic resonance imaging (MRI) of fishes housed in the Marine Vertebrate Collection (MVC) of the Scripps Institution of Oceanography (SIO). However, while we have focused on a particular imaging modality and a specific collection of specimens, the methods and integrated infrastructure, which we report here, are applicable to a wide range of applications. This is demonstrated with examples of research projects that have developed from, and the use of the techniques developed for, the DFL [8]–[15]. Moreover, the DFL infrastructure serves as one successful model for digital library creation and maintenance. It has great flexibility and can be used for data assimilation from a variety of sources, including not only newly acquired specimens from specific collections (e.g. the MVC), but incorporation of data acquired from other modalities (CT, x-ray, photography, genetics, etc), from other groups or projects, or from scanning specimens in other taxa. This digital infrastructure also provides great flexibility in data exploration.

The primary goal of the DFL was to digitize a representative set of fish species from the MVC because of the tenuous state of the future of these types of collections [1]. But beyond merely digitally preserving these specimens, digital imaging provides a means of visualizing and quantifying their anatomy, facilitating morphological investigations without the need for invasive dissection. Although there is a variety of modern high resolution scanning methods of potential utility for imaging fish, the strength of MRI is in its ability to image soft tissues, as it is sensitive to a broad range of physical parameters related to tissue physiology and architecture. For anatomical imaging, MRI may be viewed as a complimentary technique to computed tomography (CT), which has poor specificity for contrasting soft tissues but is excellent for imaging hard tissues such as bone. Both CT and MRI are currently the most widely accessible technologies with the capacity to acquire high-resolution (i.e. sub-millimeter down to around 15 µm) standardized 3D volumetric data from whole specimens encompassing the size range typical of most fishes (∼1 cm to >2 m), and have become familiar tools for imaging the in situ anatomy of animals [16]. However, the advantages of MRI come at a price - high resolution 3D MRI scanning often requires more tissue preparation in order to optimize results, scans take longer, and it is more expensive, than CT. Nevertheless, MRI has the capacity to depict detailed cross-sectional soft tissue anatomy at voxel resolutions well below 100 µm [7], [17], and is unique in its ability to not only obtain high image contrast between many different types of tissue (e.g., white and gray matter in the brain), but also measure physiological parameters such as bulk flow, perfusion, diffusion, etc [7], [17], [18].

Although MRI has previously been used for imaging fishes, beginning with Blackband et al. [19], the project outlined in this paper is a unique and broad ranging effort to develop, integrate, and standardize a complete array of imaging, visualization, analysis, archiving and dissemination methods necessary to construct a digital library with MRI data of practical use in research and education. Although fish have been scanned before, this has not been done in an optimal or standardized way since these efforts have typically been exploratory and focused on single species. Therefore we also present in detail the preparation and scanning methods that we have developed to obtain consistent high quality high-resolution data, as a step toward the standardization of MRI protocols for imaging a broad array of fish species preserved in museums.

### Anatomical MRI of fishes

There are three different contrast mechanisms that form the basis for most standard types of anatomical MRI scans: 1) the amount of water (proton density) in a voxel; 2) the MR signal recovery (or longitudinal relaxation) time, T1, and 3) MR signal decay (or transverse relaxation) time, T2 [20]. In any given voxel each of these depends on the state of local tissue microstructure, including water, fat, and protein content, etc, and thus, depending on the specific MR pulse sequence used, subtle structural differences between tissues can be made apparent [21]. However, the efficacy of MRI not only depends on the physical qualities of the scanned specimen and the software methods used for deriving tissue contrast, but also on the available MR signal-to-noise ratio (SNR) at a given voxel resolution. This is influenced by the strength of the magnetic field of the scanner and the ability of its hardware to detect the resulting MR signal in specimen tissues.

Cloutier et al. [22] were the first to apply standard anatomical MRI to postmortem fish, demonstrating that proton resonance T1, T2 and FLASH pulse sequences provided selective enhancement of cranial tissues in a coelacanth, *Latimeria chalumnae*. These methods distinguished cartilage, muscles, brain, and various connective tissues, confirming gross soft tissue anatomy previously described from dissection. Although other groups have since used similar methods to depict the internal soft tissue anatomy in a number of freshly collected and preserved species [23]–[32], resulting image quality has been limited, with the application of high quality MRI for detailed anatomical studies of fishes for comparative morphology still not sufficiently demonstrated. This has been due in part to their use of MRI scanners (typically 1.5 Tesla (T) and 3T) and hardware designed for human clinical studies, rather than those (typically 7T and 11.7T) developed for smaller animal research. Though much more widely available than high-field small animal systems, clinical scanners impose limitations on the attainable MRI signal due to their larger bore sizes (and thus lower field strengths), and restricted gradient settings necessary to ensure patient safety. Furthermore, MRI is extremely sensitive to the structural and chemical nature of tissue, and therefore the physical condition of postmortem specimens and the environment to which they have been exposed have significant impacts on imaging results. Many former studies involved the scanning of fishes that were previously frozen and thawed (e.g. [8], [9], [11], [32]), or that were exposed to alcohol in museum collections (e.g. [14], [25]–[27], [31]), processes that are known to significantly degrade tissue MRI responses, including reducing both the SNR and the contrast-to-noise ratio (CNR) [28], [33], [34]. Furthermore, with the exception of Chakrabarty et al. [14], authors did not take advantage of chemical contrast agents commonly used in clinical and biomedical MRI [35]. These agents can enhance the MR responses of tissues and improve their anatomical contrast [36], [37], and may therefore be useful for mitigating some of the adverse effects of preserving and storing fishes in museums. Optimizing methods for selecting, processing, and scanning postmortem fishes is therefore essential to enhancing the efficacy of MRI for depicting and quantifying anatomy in sufficient detail and producing consistent image quality among specimens for morphological investigation.

The goal of this paper is to present our current progress with the DFL. The DFL is a web-based platform for the archiving and retrieval of quantitative MRI data scanned from fishes preserved in the MVC. Our focus here is both on the methods employed for acquiring high field (3T and 7T) MRI data from these fishes for the purposes of digital morphology, and with our web-based methods for the visualization, analysis, cataloguing, and sharing of this data for both research and education purposes. We emphasize that the development and integration of various aspects of the DFL are an ongoing task that we hope will serve as a model for other digital biological libraries.

## Materials and Methods

### Selection and Preparation of Specimens for MRI

A critical but often overlooked aspect of scanning museum specimens using high resolution MRI is the undesirable affect that tissue preservation and storage practices can have on the MR response of tissues and on the generation of MRI artifacts, particularly at high field. For example, since MRI is based upon the behavior of water molecules, preservation methods that reduce the amount of tissue water and lipids can significantly reduce the available MR signal and contrast of tissues, as well as alter the physical structure of tissue itself, i.e. via shrinkage. While specimens do not necessarily require any preparation for MRI, and in fact museum fishes have even been imaged directly inside their alcohol storage jars [27], image quality can be significantly enhanced if specimens undergo some degree of sample preparation prior to scanning. Therefore, in order to mitigate undesirable image artifacts and increase the quality and uniformity of imaging results among specimens, we have developed a standardized protocol for preparing MVC fishes for MRI. However, the utilization of these methods with specific specimens is at the discretion of collection managers.

Fish specimens were obtained from the MVC where they are typically fixed in 10% formalin and post-fixed in 50% isopropyl alcohol for long-term storage. A small number of additional specimens were acquired from outside institutions where they were typically post-fixed in 70% ethyl alcohol. A number of newly acquired MVC specimens were also made available for MRI directly following formalin fixation. Intact, straight-bodied, adult fishes with minimal signs of damage were preferentially selected for imaging, with the exception of very large species where juvenile specimens of a more practical size for our RF coils were obtained. Specimens were removed from storage and carefully rehydrated in successive reductions of 75%, 50%, and 25% alcohol over a period of days, followed by full rehydration in water with the addition of the biocide, sodium azide (0.01%). This allowed alcohol to be cleared from tissues without damaging the specimen. Fish acquired directly following formalin fixation were washed in successive exchanges of water to remove unbound fixative prior to scanning. Specimens small enough to be imaged on the 7T scanner were then soaked for a minimum of two additional weeks in a solution of phosphate buffered saline (PBS) with 0.01% sodium azide and 2.5 mM of the gadolinium-based MR contrast agent ProHance (gadoteridol: Bracco Diagnostics, Princeton, NJ) (modified from [38]). This step was not practical for larger specimens due to the currently prohibitive cost of gadolinium-based contrast agents and limitations achieving even diffusion of this agent into large volumes of tissue within reasonable time. 7T specimens were securely taped onto an acrylic platform that was placed inside a 65 mm internal diameter acrylic canister filled with the perfluoropolyether fluid, Galden (Solvay Solexis, Houston, TX). The canister was then sealed and lightly pressurized to reduce or eliminate pockets of air that may cause artefacts in T1-weighted images. 3T specimens were wrapped in plastic sheeting in order to keep them moist and secured with tape to a rigid acrylic platform prior to scanning.

### MRI Data Acquisition

MRI data were acquired with either of two scanners housed at the Keck Center for Functional Magnetic Resonance Imaging (CFMRI) at UCSD. These included a 3T (127.7 MHz) human clinical scanner (Signa Excite 750; GE Healthcare, Milwaukee, WI) equipped with a 55 cm bore with full 45 cm field-of-view (FOV) imaging capability and gradient strengths 4.4 mT/m (across the bore), with a maximum slew rate of 250 mT/m/ms and a rise time of 150 µs, and a 7T (300 MHz) small animal scanner (Bruker Biospec Avance II, Bruker AXS Inc., Madison, WI), consisting of a 210 mm horizontal bore magnet equipped with a shielded gradient set with an inner diameter of 90 mm, and a maximum gradient strength of 630 mT/m, maximum slew rate 6300 T/m/s, and rise time 160 µs. Fish with body diameters larger than 65 mm were imaged on the 3T scanner using standard radio-frequency (RF) coils, including an 8-channel torso coil, 8-channel cardiac coil, or 8-channel head coil (MRI Devices, Waukesha, WI), the choice of which depended on specimen size and shape. As a general rule, the RF coil with the greatest number of coil elements (channels) and of the smallest diameter necessary for encompassing the intact specimen typically provided the best SNR. Smaller fish with diameters 65 mm or less were imaged on the 7T scanner using either a 35 mm or 72 mm inner diameter quadrature RF volume coil (Bruker Biospin GmbH, Ettlingen, Germany), the choice of which also depended on fish size. High-resolution isotropic images were acquired with standard T1-weighted 3D fast spoiled gradient recalled echo (FSPGR) acquisition pulse sequences [21]. For each specimen it was necessary to optimize image contrast since MR tissue responses were essentially unique due to variations in their preservation histories. Thus the specific parameters used for image acquisition varied slightly among specimens due to differences in their size and shape, and the condition of their tissues. Specimens imaged on the 3T scanner typically used the following pulse sequence parameters: 30–35° flip angle (FA), 8.2–11.9 ms repetition time (TR), 2.3–3.9 ms echo time (TE), 127 kHz bandwidth, and 4 averages. Images were collected in the transverse plane at isotropic voxel resolutions between 350–700 µm^3^, encompassing slice thicknesses between 300–700 µm, and a unique in-plane field of view (FOV) and slice matrix for each specimen. Scan times were typically between 20–40 minutes per volume. 7T specimens were typically imaged with the following pulse sequence parameters: 15° FA, 20–30 ms TR, 10–15 ms TE, 300 kHz bandwidth, and 3–4 averages, with images collected in the transverse plane at an isotropic voxel resolution of 100 µm^3^. This encompassed a slice thickness of 100 µm with a unique in-plane FOV and slice matrix for each specimen, and scan times of approximately 90–120 minutes per volume. Specimens that exceeded the FOV of available RF coils (e.g. >120 mm SL for 7T scanning; >280 mm SL for 3T scanning) were scanned in multiple, overlapping volumes and pieced together using image registration methods (see below). All specimens were digitally photographed for the DFL before being rehydrated and returned to the MVC.

### MRI Data Processing and Archiving

Raw data from MRI scanners is digital, and is thus easily stored in both its original (original 32-bit integer) format as well as in any number of commonly accepted standardized medical imaging formats. DFL data are stored both in raw and standard medical imaging DICOM (http://medical.nema.org/) and NIfTI (http://nifti.nimh.nih.gov/) formats which are commonly accepted formats for numerous image registration, segmentation, visualization and image analysis programs. These data are archived in the DFL database for public access via the DFL website (http://www.digitalfishlibrary.org/library/). As described in more detail below, the DFL database was designed to accommodate a number of functions useful for investigating morphology. This includes remote archiving and retrieval of 3D MRI slice and segmentation data and visualizations, information on scanning parameters, field collection data, species taxonomic hierarchies and general biology, links to other relevant databases, as well as our prototype online data visualization module, the DigiFish Viewer. In order to keep track of how our data is used, which is a requirement for the use of MVC collection specimens, the DFL has been set up to function like a standard library or collection, in that users must register in order to check out data. We developed registration and security protocols to do this, and we regularly interact with outside users to provide data. However, to protect the priority of an author, specimens scanned as part of a collaborative research project are not made available until after the publication of the study.

MRI data for specimens collected in multiple image volumes were merged into single datasets using a mutual information algorithm feature in Amira (Visage Imaging Inc, San Diego, CA). This image registration method maximizes the joint probability distribution between two images to bring them into alignment. Image segmentation of selected soft tissue structures was performed using ITK-SNAP (Insight Segmentation and Registration Toolkit) [39]. Image segmentation is the process of digitally defining separate tissues and organ systems from volumetric image data. These 3D segmented regions then exist as separate entities that can be displayed (and rotated, zoomed, etc), and quantitatively analyzed independently of one another. Most segmentation by slice was performed using a combination of semi-automatic and manual image segmentation tools. However, segmentation of MRI data, unlike CT data, is extremely time-consuming and was therefore performed on a selection of internal organs in only a small subset of DFL specimens to date.

Movies and jpeg images of MRI slice plane data were created in ImageJ (http://rsbweb.nih.gov/ij/). QuickTime virtual renderings (QTVR) of 3D image data were created using OsiriX (http://www.osirix-viewer.com). QuickTime movies of 3D segmented data were generated using the Visualization Toolkit (VTK) (http://www.vtk.org/). All QuickTime content was converted to Adobe Flash format using FFMpeg (http://ffmpeg.org). Labeling of soft tissue structures on MRI jpeg images was performed in Adobe Photoshop CS2 9.0. Note that, since most of our MRI datasets were significantly less than 1 GB (typically between ∼100–500 MB), image processing and data analysis were readily handled with current desktop PC capabilities.

## Results

### Optimizing Preparation and Scanning of Museum Specimens

**Figure 1:**
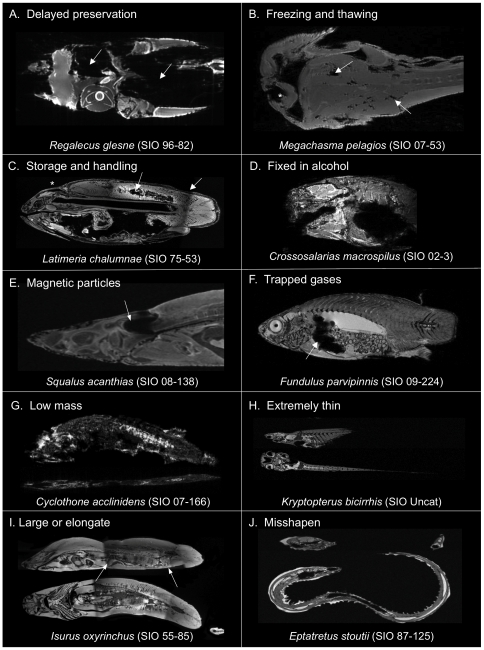
Common problems encountered when scanning museum fishes. Specimens were prepared for imaging and scanned as described in the Methods. Specific details for each species can be found in the DFL. (A) 3T horizontal image slice (937 µm^3^ resolution) of an oarfish, *Regalecus glesne* (SIO 96-82; SL 730 cm) demonstrating the effects of delaying soft tissue preservation. Arrows indicate tissues with significant decomposition. (B) 3T horizontal image slice (1.1 mm^3^ resolution) of a megamouth shark, *Megachasma pelagios* (SIO 07-53; standard length (SL) 215 cm) with significant tissue damage (arrows) from repeated freezing and thawing prior to fixation, resulting in poor tissue MRI signal and contrast. The asterisk indicates mechanical damage to the jaw. (C) 3T sagittal image slice (761 µm^3^ resolution) of a coelacanth, *Latimeria chalumnae* (SIO 75-347; SL 950 mm) demonstrating poor MRI results from being frozen and thawed prior to fixation and from extensive handling including dissection. (D) 7T sagittal slice (100 µm^3^ resolution) of a triplespot blenny, *Crossosalarias macrospilus* (SIO 02-3; SL 73 mm) exhibiting poor imaging results caused by exposure to alcohol without prior formalin fixation. (E) 3T sagittal slice (586 µm^3^ resolution) of a spiny dogfish, *Squalus acanthias* (SIO 08-138; SL 740 mm) showing the presence of inorganic particles in the inner ear causing a prominent magnetic susceptibility artefact. (F) 7T sagittal slice (100 µm^3^ resolution) of a California killifish, *Fundulus parvipinnis* (SIO 09-224; SL 68 mm) exhibiting a prominent magnetic susceptibility artefact caused by gases trapped in its gut. (G) 7T sagittal and horizontal slices (60 µm^3^ resolution) of a benttooth bristlemouth, *Cyclothone acclinidens* (SIO 07-166; SL 53 mm) exhibiting very poor image contrast caused by its extreme low body mass leading to an insufficient signal loading of the RF coil. (H) 7T sagittal and horizontal slices (100 µm^3^ resolution) of a glass catfish, *Kryptopterus bicirrhis* (SIO Uncat; SL 45 mm) showing problems with slice plane misalignment associated with scanning very flat or thin-bodied fishes. (I) 3T sagittal and horizontal slices (586 µm^3^ resolution) of a shortfin mako, *Isurus oxyrinchus* (SIO 55-85; SL 875 mm) showing slice plane misalignment problems associated with scanning large and/or elongate specimens requiring sequential repositioning of the coil to scan their full length. (I) 7T sagittal and horizontal slices (100 µm^3^ resolution) of a Pacific hagfish, *Eptatretus stoutii* (SIO 87-125; SL 145 mm) illustrating slice plane misalignment problems associated with scanning specimens preserved with body positions that cannot be straightened, preventing acquisition of bilaterally symmetrical slices in every plane.

**Figure 2:**
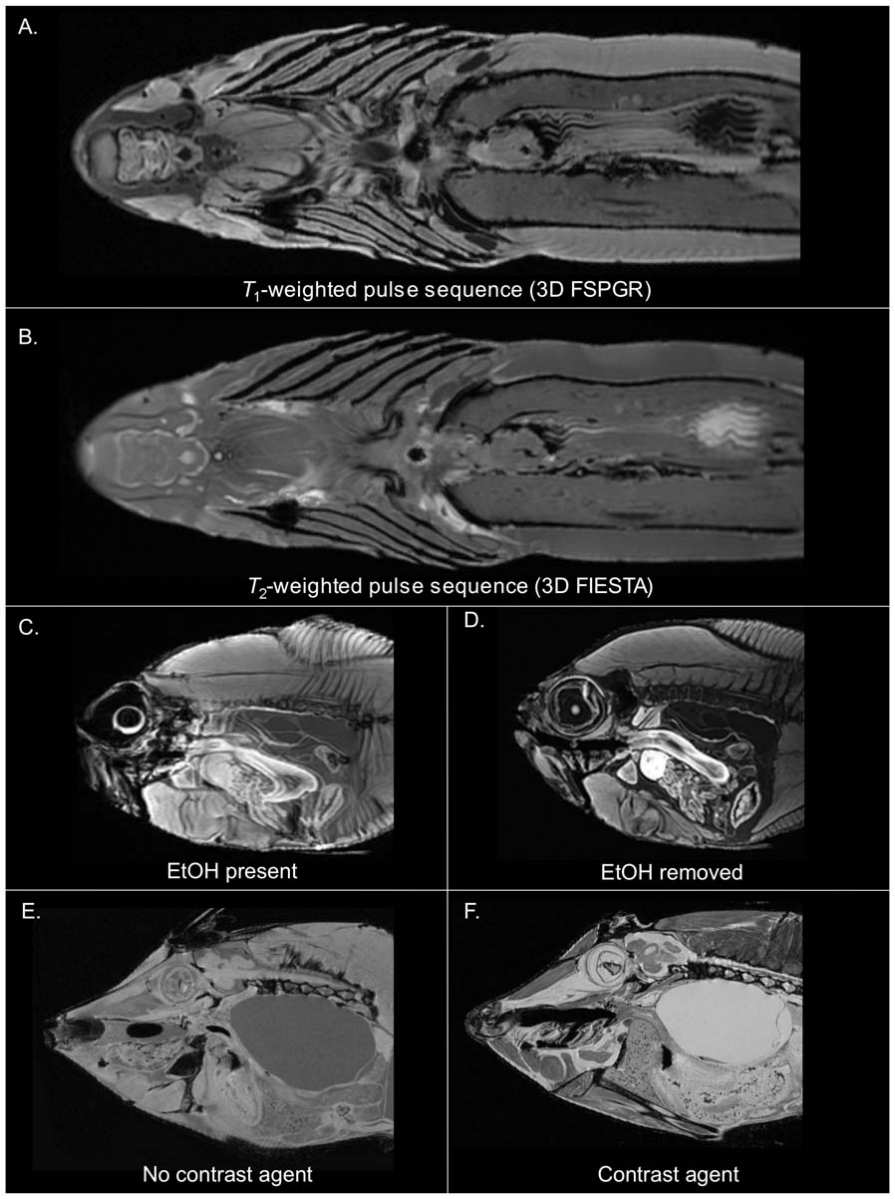
Optimizing preparation and scanning of museum specimens. (A) Comparison of T1-weighted 3D FSPGR (fast-spoiled gradient-recalled echo) and (B) T2-weighted 3D FIESTA MRI pulse sequences acquired on the 3*T* scanner (slice matrix = 512×512×236, slice thickness = 900 µm, resolution = 683 µm^3^, averages = 2) in the smooth hammerhead, *Sphyrna lewini* (SIO 64-528, SL 104 cm). Additional parameters for the T1-weighted 3D Fast-Spoiled Gradient Echo (FSPGR) pulse sequence include, FA = 35°, TR = 9.86 ms, TE = 4.112 ms, and for the T2-weighted 3D FIESTA pulse sequence, FA = 40°, TR = 4.456 ms, TE = 2.1 ms. (C) The red bream, *Beryx decadactylus* (SIO 85-77; SL 289 mm), was initially imaged with a T1-weighted FSPGR pulse sequence on the 3*T* scanner prior to rehydration, and (D) re-imaged following rehydration resulting in an enhanced image quality. Scan parameters: FA = 30°, TR = 11.904 ms, TE = 3.932 ms, slice matrix = 512×512×236, slice thickness = 600 µm, resolution = 527 µm^3^, averages = 3. (E) The fantail filefish, *Pervagor spilosoma* (SIO 53-539; SL 74 mm) was initially imaged on the 7*T* scanner using a T1-weighted FLASH pulse sequence without exposure to contrast agent, ProHance. (F) It was subsequently reimaged following exposure to 2.5 mM ProHance, resulting in a significantly brighter MR signal and enhanced visual contrast among tissues. Scan parameters: FA = 15°, TR = 25.875 ms, TE = 12.853 ms, slice matrix = 350×1000×420, slice thickness = 100 µm, resolution = 100 µm^3^, averages = 8.

Most fishes imaged for the DFL were acquired by the MVC for purposes other than MRI and thus their tissues were not preserved using protocols optimized for high resolution MRI. Figures 1 and 2 illustrate some specimen and imaging attributes that assisted in optimizing the criteria we choose for selecting and preparing these specimens for MRI, and in our subsequent choices of scanner hardware, pulse sequence, and scan parameters. Note that in these images voxels of hypo-intense black indicate tissues with minimal water or lipid content, and voxels of hyper-intense white indicate tissues with maximal water or lipid content. Also, since the T1 of muscle in preserved fishes is approximately double that of fat [28], muscle characteristically appears darker in contrast to more fatty tissues such as those of the brain and liver.

Figure 1 (A–J) presents T1-weighted images of specimens exhibiting a range of physical attributes that were frequently found to impair the results of MRI and segmentation. Figure 1A clearly shows the effects of delaying tissue preservation, with this oarfish (*Regalecus glesne*) sustaining substantial decomposition and loss of soft tissues (arrows) by the time it was retrieved for preservation, greatly reducing its viability for MRI. Figure 1B illustrates the adverse effects that multiple freezing and rapid thawing events have on specimens, with this megamouth shark (*Megachasma pelagios*) sustaining significant soft tissue degradation (arrows) from poor handling and the extensive loss of water prior to chemical preservation. This has resulted in obvious damage to some anatomical structures and the inevitable loss of MR signal and contrast apparent in this image. Figure 1C presents MRI data illustrating the adverse affects that freezing and thawing, and in addition, long-term storage in alcohol, can have on specimens. In this image of the coelacanth (*Latimeria chalumnae*), the brain has visibly degraded (asterisk) and the muscles of the trunk have a ‘flaky’ appearance (arrow) indicating extensive tissue shrinkage and degradation over the 40 years or more that it has been preserved in the collection. More importantly, this specimen has also been partially dissected with most of the internal organs located in its abdominal cavity having been removed. Figure 1D illustrates the effects that preserving specimens directly in alcohol can have on MRI quality. To retain viable DNA for molecular analysis, this triplespot blenny (*Crossosalarias macrospilus*) was preserved directly in 50% isopropyl alcohol. This not only promoted the shrinkage and distortion of its soft tissues, but also a significant reduction in available MRI signal and contrast caused by the rapid and extreme loss of water and lipids from the unfixed specimen. Figure 1E reveals the presence of ferromagnetic particles of magnetite in the inner ear of this spiny dogfish (*Squalus acanthias*). Ferromagnetic particles interfere with the magnetic field of the scanner, producing a ‘blooming’ susceptibility artefact (arrow) that can mask underlying anatomy. Note that similar effects were also observed in specimens that had metallic objects such as a fishing hook lodged in their guts. Figure 1F demonstrates the effects that trapped gases (arrow) have on T1-weighted images at 7T. Trapped gases of undetermined origin can be seen within the body cavity of this California killifish (*Fundulus parvipinnis*), producing a prominent susceptibility artefact that masks underlying anatomy. In this case, T2-weighted imaging is a viable alternative since it is less vulnerable to this imaging artefact. Figure 1G presents an example of imaging small specimens with extremely low body mass such as this benttooth bristlemouth (*Cyclothone acclinidens*). Although the specimen was in good condition and the correct selection of scanner hardware and image acquisition methods were made, the specimen did not provide an adequate signal load for the coil. As a result, MR signal and contrast were not sufficient to adequately visualize soft tissue anatomy. This problem can be mitigated in most instances by scanning the specimen partially, or fully, immersed in water to increase proton and hence signal availability. Figures 1H–J highlight some common problems associated with specimen body shape. Bilaterally symmetrical slices are difficult to acquire in very flat or thin-bodied fishes such as the glass catfish (*Kryptopterus bicirrhis*), with data often requiring re-slicing during post-processing in order to obtain datasets with symmetrical slice planes. Large and/or elongate specimens such as this shortfin mako (*Isurus oxyrinchus*) (Figure 1I) have body lengths that exceeded the FOV of our available RF coils, requiring sequential repositioning of the coil along the body in order to scan the entire specimen. In this example, misalignments of the coil had occurred (arrows), requiring datasets to be re-sliced during post processing in order to correctly align the anatomy of overlapping slices. Also, the insufficient overlap of sections did not allow the removal of slice data acquired near the edges of the coil where signal strength is reduced. Hence, distortions in the images are apparent, particularly in the sagittal plane. Figure 1J illustrates the issue associated with scanning specimens that straight when their tissues were fixed, as was the case with this Pacific hagfish (*Eptatretus stoutii*). This prevents acquisition of bilaterally symmetrical slice planes that the re-slicing of image datasets cannot correct.

Figures 2A and 2B show results typical of T1-weighted imaging versus T2-weighted imaging of preserved fishes. In this representative example using the smooth hammerhead (*Sphyrna zygaena*), the T1-weighted pulse sequence (Figure 2A) provided greater MR contrast among different tissue types than the T2-weighted pulse sequence (Figure 2B), and thus enhanced differentiation of major soft tissue structures. As a result, anatomical details of the brain, sense organs, musculature, alimentary tract, etc, were more apparent, with cartilage particularly well contrasted, thereby aiding anatomical identification, segmentation, and morphological analysis. A T1-weighted pulse sequence was therefore used as the primary pulse sequence for acquiring general anatomical data for the DFL on both 3T and 7T scanners. Figures 2C and 2D illustrate the increase in image quality achieved by clearing post-fixative alcohol from specimens prior to scanning. In this example, a red bream (*Beryx decadactylus*) was removed from 50% isopropyl alcohol, soaked briefly in PBS to reduce the amount of alcohol pooled within its body cavities, and imaged using a T1-weighted sequence on the 3T scanner (Figure 2C). The presence of alcohol within its tissues produced a strongly iso-intense image artefact as evidenced by the ‘blurriness’ of the image. Alcohol was subsequently cleared from the specimen (see Methods) and it was rescanned using identical image acquisition methods. This significantly increased image sharpness and contrast (Figure 2D). Figures 2E and 2F show enhancements in T1-weighted 7T imaging results achieved with the use of a gadolinium-based contrast agent. In this example, a fantail filefish (*Pervagor spilosoma*) was initially scanned following the clearance of alcohol from tissues (Figure 2E). It was then exposed to Prohance (see Methods) and rescanned using identical image acquisition methods. This resulted in a significant increase in image brightness and SNR, and an overall enhancement in the visual contrast among different soft tissues, including a clearer delineation of brain structures, features of the alimentary tract, the liver, the heart, muscles of the head and body, as well as an increase in the brightness of fluid-filled spaces such as that of the swim bladder, eye, and intracranial cavity (Figure 2F). These improvements had significant benefits for the efficiency of image segmentation. For example, the semiautomatic segmentation algorithms in ITK-SNAP functioned more successfully on contrast enhanced tissues.

### Curated MRI Data

**Figure 3:**
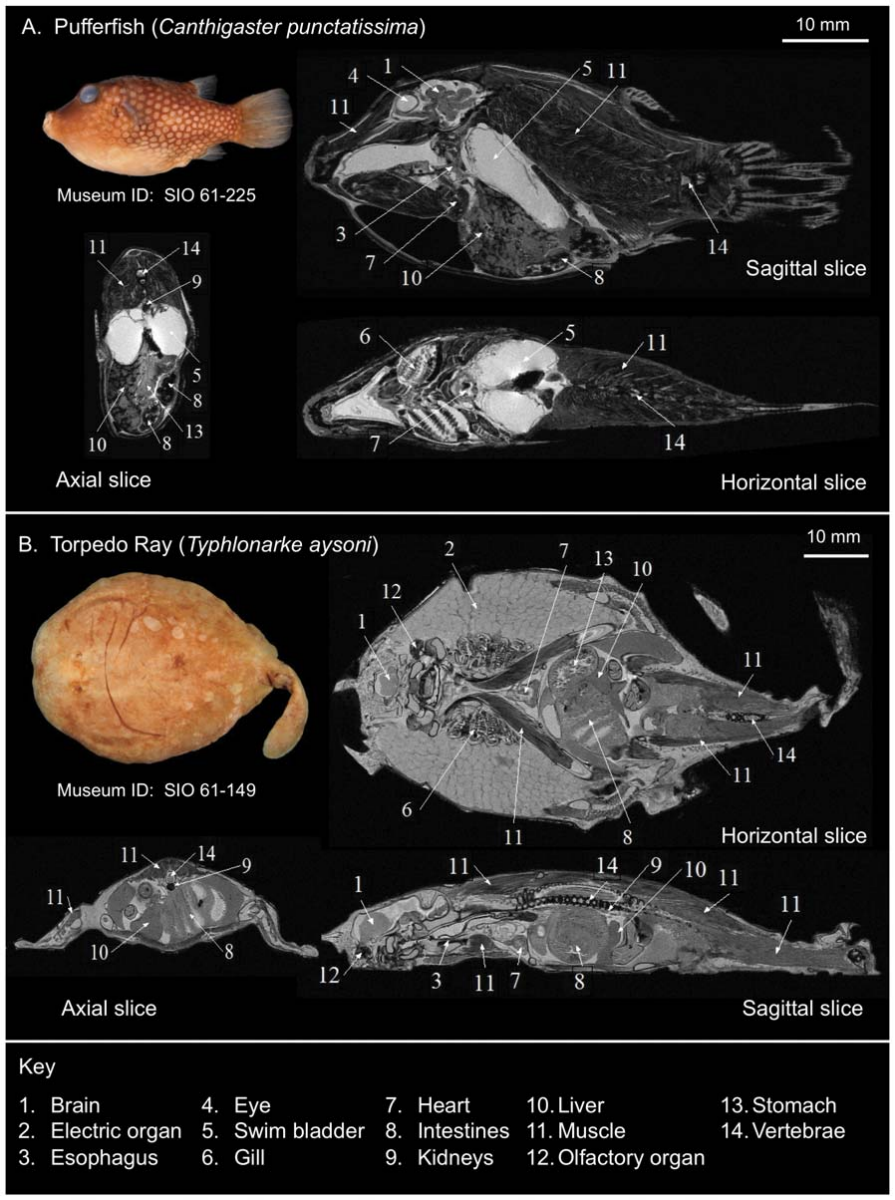
Examples of T1-weighted contrast-enhanced 7T MRI data. (A) Spotted sharpnose puffer, *Canthigaster punctatissima* (SIO 61-225, SL 57 mm). Pulse sequence parameters include: FA = 15°, TR = 22.814 ms, TE = 11.322 ms, slice thickness = 100 µm, slice matrix = 354×780×210, resolution = 100 µm^3^, averages = 5. (B) Blind legged torpedo, *Typhlonarke aysoni* (SIO 61-149, SL 92 mm). Pulse sequence parameters include: FA = 15°, TR = 23 ms, TE = 11.222 ms, slice thickness = 100 µm, slice matrix = 550×830×180, resolution = 100 µm^3^, averages = 3. Numbered labels indicate a selection of readily visible anatomical structures in these slices.

Our standard specimen processing and T1-weighted imaging protocols have allowed the acquisition of high resolution, high contrast 3D anatomical MRI data for over 300 MVC specimens currently archived in the DFL, including at least one representative species from 195 of the 515 families, and 56 of the 62 orders, described in Nelson [40]. As illustrated in Figure 3, these methods produce anatomical images with sufficient contrast to allow the identification and segmentation of a broad selection of soft tissue structures in both bony and cartilaginous fishes. These structures typically include: skeletal cartilages and muscles; circulatory system structures including the heart and major arteries; digestive system structures including the esophagus, stomach, intestines, pancreas, liver and gall bladder; urinary system structures including the kidneys; nervous system structures including the brain, large cranial and peripheral nerve tracts, sense organs including eyes, inner ear, lateral line, and olfactory organs; as well as fluid-filled spaces including the intracranial space, those of the eye, abdominal space, and the swim bladder (which typically become inundated with fluid during preservation). Although not depicted here, reproductive structures containing mature gametes, and even developing embryos, were also visible in some specimens. Gut contents were also found in a number of others. Although bony tissue has much lower MR signal intensity relative to soft tissues, the fatty and collagenous matrix that typically surrounds vertebral bone often provides it with good contrast, as observed in Figure 3A. This is owing to the shorter T1 value of the lipid-containing (i.e. fatty) tissue with respect to the water-containing (i.e. collagenous) tissue, thus enhancing its visibility. Overall, the resulting MRI data facilitates the quantitative analysis of segmented anatomy using a multitude of image analysis tools, including those available in ITK-SNAP and Amira, and our DigiFish Viewer.

### 3D Segmentation and Visualization

**Figure 4:**
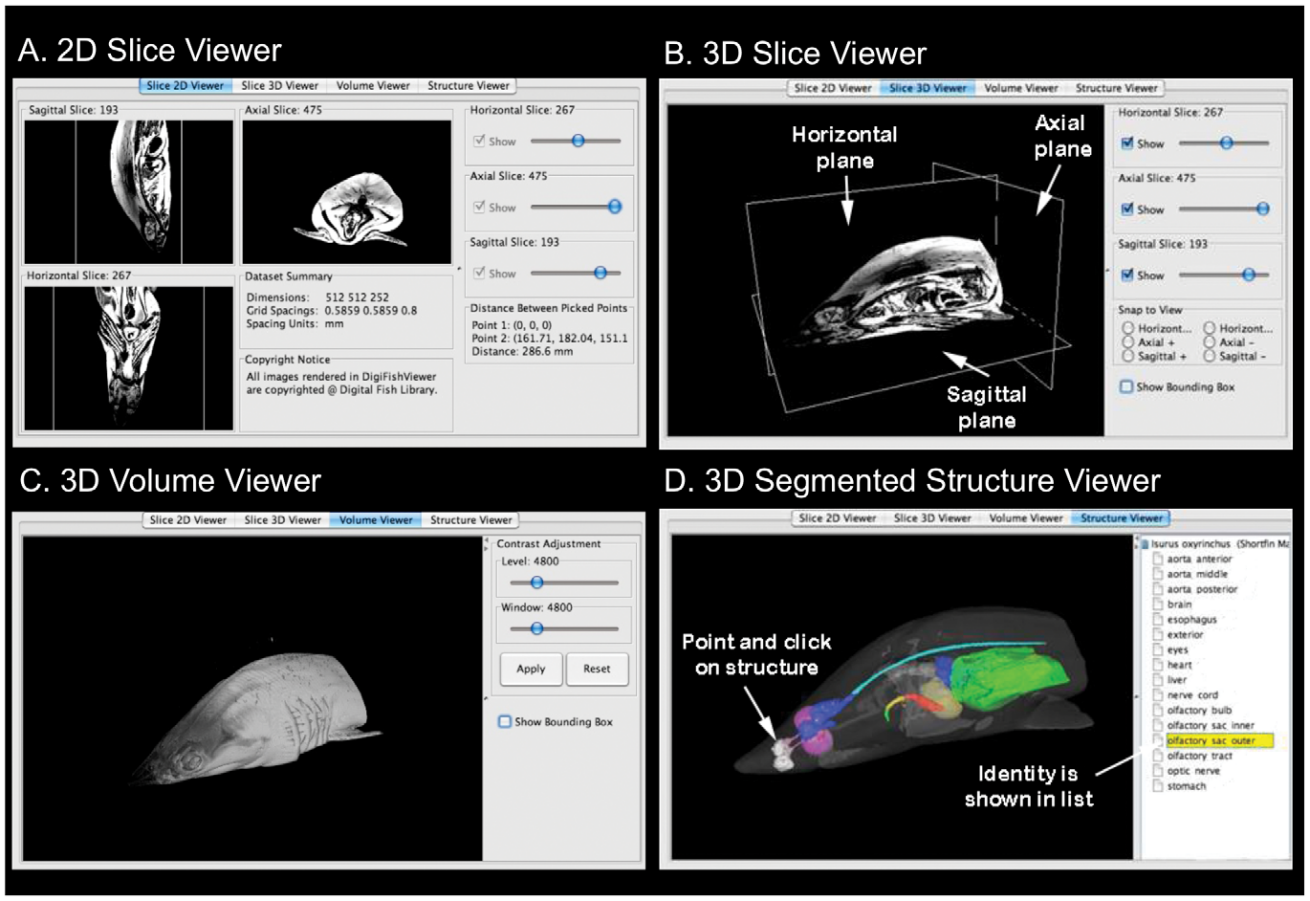
DigiFish Viewer with shortfin mako (*Isurus oxyrinchus*) MRI data. (A) 2D Slice Viewer displays slices in each orthogonal plane. Distances between points within slices can be measured. (B) 3D Slice Viewer displays an intersecting slice from each orthogonal plane in a single view that can be reoriented in 3D. (C) 3D Volume Viewer displays slice data as a 3D volume. Image contrast can be adjusted which also takes effect in the Slice Viewers. (D) 3D Segmented Structure Viewer displays 3D renderings of segmented structures. In this example, the skin (rendered partially transparent) and a selection of internal organs are shown. The olfactory sacs have been selected from the list of available structures and are highlighted (in white) in the viewer window.

The DigiFish Viewer was developed as a prototype platform for interactive viewing of our NIfTI formatted MRI datasets and VTK segmented data online via the DFL website (Figure 4). It is a Java application built and deployed using Java Web Start and Java Network Launch Protocol technology (http://java.sun.com/javase/technologies), allowing the DigiFish Viewer to run independent of user operating system and drivers. The basic requirement is the latest Java runtime environment and installation of Java3D software (http://java.sun.com/javase/technologies/desktop/java3d/). The DigiFish Viewer graphical user interface is initiated through the browser using an icon located on each species page and offers four data display modes. The 2D Slice Viewer (Figure 4A) depicts each of the three 2D slice planes in an independent window, each of which can be scrolled slice by slice. This display mode also features a measurement function where the distance (in mm) between any two points within a window can be calculated. The 3D Slice Viewer (Figure 4B) displays all three slice planes in the same 3D environment while allowing each to be scrolled or visualized independently, as well as rotated, zoomed, or panned. The 3D Volume Viewer (Figure 4C) renders the 2D slice data into a 3D volume, which can also be rotated, zoomed, and panned. This display mode also provides an image contrast adjustment feature. The 3D Structure Viewer (Figure 4D) displays the available segmented volumetric data. Along with rotate, zoom, and pan, individual structures can be selected within the 3D object window and their identity highlighted within a list of available structures. Conversely, the name of a structure can be selected from within the list with its associated 3D rendered object highlighted. However, contrary to expectations, changes to Java runtime environments from automatic software updates on the personal computers of users may introduce incompatibilities with the current version of the viewer, breaking this functionality for some users. We aim to resolve these software conflicts for all users in the near future. Despite this, the prototype DigiFish Viewer has provided a useful demonstration of the utility of investigating 3D datasets over the web using simple tools. As with all DFL data, users can request our segmented datasets for download and use with any image processing software of their choosing.

**Figure 5:**
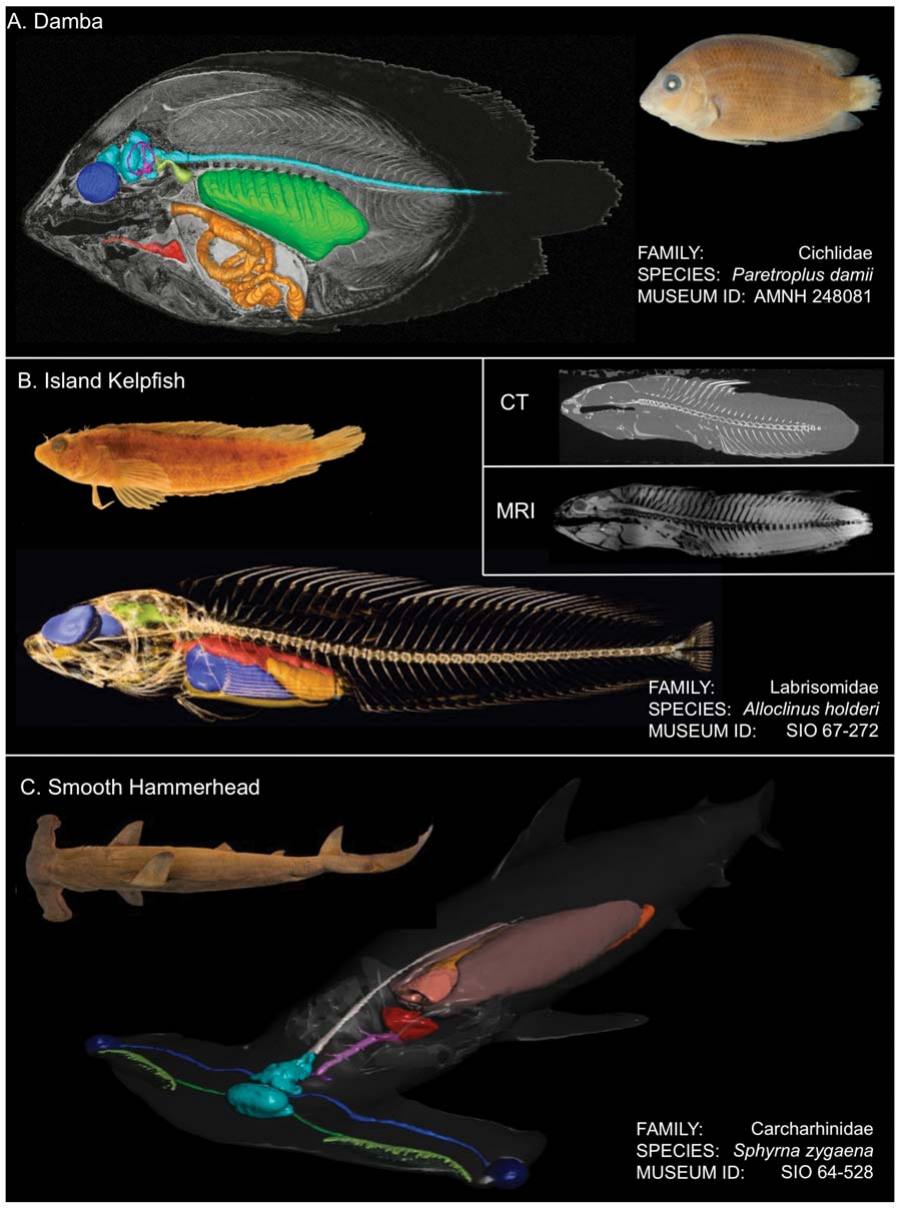
Examples of 3D segmentation and rendering of DFL data. (A) This damba, *Paretroplus damii* (AMNH 248081, SL 76 mm) was imaged at 7*T* with the following 3D FLASH pulse sequence parameters: FA = 15°, TR = 24.07 ms, TE = 11.92 ms, slice thickness = 100 µm, slice matrix = 300×1024×500, resolution = 100 µm^3^, averages = 2. MRI slice data is overlaid with a selection of segmented soft tissue structures color-coded as follows: light blue = brain and spinal cord; dark blue = eyes; magenta = vestibular labyrinth; green = gas bladder; light brown = alimentary tract; red = heart and dorsal aorta. (B) Both MRI and CT data were acquired for this island kelpfish, *Alloclinus holderi* (SIO 67-272, SL 94 mm). A selection of soft tissue structures were segmented from MRI data and are color-coded as follows: blue = eyes; green = brain; red = alimentary tract; light brown = ovaries; purple = liver. Segmented soft tissues were co-registered with a volumetric rendering of CT imaged hard tissue structures acquired by The University of Texas High-Resolution X-ray Computed Tomography Facility (UTCT) (slice thickness 42 µm). (C) This smooth hammerhead, *Sphyrna zygaena* (SIO 64-528; SL 104 mm) was imaged at 3*T* with the following 3D FSPGR pulse sequence parameters: FA = 35°, TR = 9.86 ms, TE = 4.11 ms, slice thickness = 900 µm, slice matrix = 512×512×236, resolution = 683 µm^3^, averages = 2. It was scanned in 4 sections and pieced together in Amira for segmentation. A selection of segmented soft tissue structures are color-coded as follows: brown = alimentary tract; dark blue = eyes; green = olfactory system; light blue = brain; magenta = dorsal aorta; puce = liver; red = heart; white = spinal cord.

Figure 5 presents results for three segmented DFL species, including the damba (*Paretroplus damii*) (Figure 5A), island kelpfish (*Alloclinus holderi*) (Figure 5B), and smooth hammerhead (*Sphyrna zygaena*) (Figure 5C). Note the island kelpfish (Figure 5B) also includes CT data depicting bony tissues that were subsequently co-registered with the MRI dataset. This figure highlights the differences between these modalities in terms of the relative contrast of hard and soft tissues. In the CT image, bone (white) is brightly contrasted against more poorly contrasted soft tissues (grey), providing excellent definition of skeletal structures but very limited soft tissue differentiation. In the T1-weighted MRI image, soft tissues (grey) have much greater contrast than in the CT image, while the presence of bone may be inferred from voxels where there is minimal or no signal (black). Overall, the imaging and segmentation methods that we employ allow the creation of anatomically accurate 3D digital models depicting the relative size, shape, and position of soft tissue structures in preserved fish. The segmented 3D models and the MRI datasets from which they were derived can be viewed interactively on the DFL website with the DigiFish Viewer (Figure 4) and are also available to users for download.

### DFL Database and Website and Data Dissemination

The DFL relies on a collection of open-source software including a database (MySQL), a flexible development framework (QCodo), and the web server itself (Apache). The DFL website features a combination of HTML, Adobe Flash, and JavaScript. The database was built using MySQL and was designed with a tree structure that parallels the taxonomic hierarchy of fishes from class down to species level. A PHP-based tool was used to handle the administration of MySQL over the web, including various tasks such as creating, modifying or deleting database entries, tables, fields, or rows; executing SQL statements; displaying database contents; and managing users and permissions. A QCodo Development Framework (http://www.qcodo.com/) was used on top of MySQL and PHP to facilitate the creation of PHP objects based off MySQL database tables. The database comprises numerous fields and objects, including MRI data types, multimedia content, taxonomic hierarchies, text, and HTML links. The front-end of the database is the DFL website which facilitates the curation and display of all content on the server. Content can be uploaded, edited, and retrieved remotely using an administrative management feature on the website called MyDFL. This framework is highly robust and scalable, and enables the creation of new features with little or no interruption to existing functions.

The DFL database and website together facilitate the online organization and display of our large, complex MRI voxel data sets and associated metadata. Users allocated a particular type of user account can remotely modify the database content and access full datasets through the MyDFL feature on the website. DFL accounts have been completed for all imaged specimens along with information about their families, orders, and classes. The following content is included for each archived specimen: taxonomic classification (species, family, order, and class); a high-resolution specimen photograph; a link to its entry in the MVC online catalogue (http://collections.ucsd.edu/mv/fish/frm_search.php); specimen morphometrics; field collection information; movies and images of MRI slice data, volume renderings, and segmented structures; MRI scan and image acquisition information; and general biological information compiled from the literature detailing species morphology, ecology, physiology, and behavior as well as that of its family, order, and class. Links are also provided to species entries in other online databases, such as FishBase (http://www.fishbase.org/) and the Encyclopedia of Life (http://www.eol.org/). For a growing number of specimens, annotated images of select MRI cross-sections and segmented renderings showing the identity of particular soft tissue structures have also been uploaded, providing the first instance of anatomical data available in many of our imaged species. The library can be searched using various methods, including by: 1) keyword, 2) an interactive cladogram-style Flash animation displaying imaged species by their taxonomic relationships, 3) tiled thumbnails displaying MRI slices, segmented renderings, and specimen photos, and 4) a “Quick Find” menu organized by available species, families, orders, and classes. The DFL website also includes pages outside of the specimen library that feature additional content developed for public outreach (e.g., http://www.digitalfishlibrary.org/education).

Data dissemination is structured so that anyone with web access can request DFL data. Due to the requirements of monitoring MVC specimen usage, in conjunction with well-established web security compliances, user access requires a request to the DFL administration that outlines the intended use of the materials. A secure user account is then established and enables downloading of the MRI data. Our goal is to create a digital library that functions as a standard library or collection: users must register and check out items. However, we recognize that there are subtleties to data dissemination and the resolution of issues is evolving with the increasing development and usage of digital libraries. For example, in order to protect the priority of authors, DFL specimens scanned as part of a collaborative research project are not made publicly available until after the publication of the data. Registration and security protocols have been developed to do this. We regularly interact with outside users to provide data and it is our hope that the protocols that we have established will serve as a model for the development and usage of other digital libraries.

## Discussion

Although the molecular revolution in phylogenetic biology has raised significant challenges to evolutionary hypotheses based on morphology [41], the analysis of morphological variation remains at the heart of our understanding of biodiversity and the evolution of biological form and function [42]. Dating back to the time of Aristotle, documentation and illustration of anatomy has been fundamental to the study of comparative morphology [43], with its role revitalized from time to time as data from various research fields are integrated [44] and as new methods of data acquisition (e.g. [4], [45], [46]), and analysis (e.g., [47]–[49]) arise. Recent technological advances have led to digital imaging becoming a familiar and versatile part of modern biology [50]. Although high-throughput, non-invasive digital imaging devices such as MRI and CT scanners are relatively expensive and thus only sporadically used in comparative morphology, they are proving to be powerful tools for the 3D quantification and analysis of anatomical structure in the context of the whole organism [16], [51]–[57]. CT and MRI are unhampered by limitations relating to the depth of signal penetration and therefore do not necessarily require any special preparation of specimens prior to imaging. This has great advantages where the dissection of specimens is not desired, as is frequently the case in museum collections [14], [25], [52], or even possible where rare or endangered species are involved [57]. These technologies also offer an efficient method for permanent storage of large volumes of quantitative digital data that requires very little physical space, promotes the use of sophisticated data analysis and visualization methods, and allows the dissemination of this data online in standard formats [5], [50].

### Specimen Preparation and Imaging

Although our high-field T1-weighted MRI methods have been successful in visualizing and segmenting soft tissue anatomy in a broad array of MVC fishes, thereby reducing the need for dissecting specimens, one of the most critical factors that have emerged in the DFL project is the importance of the specimen preparation process. Scanning preserved fishes, rather than live or fresh post-mortem specimens, significantly enhances attainable image quality, as it stabilizes tissues and produces datasets devoid of motion, facilitating much longer scan times with higher achievable SNR, and thus considerably higher image resolutions. However, image quality can be compromised when scanning specimens that have not been optimally preserved for MRI, as is the case for virtually all fish collection specimens. Since the magnitude of the MR signal depends on the local state of tissue water and its surroundings, the fixation and preservation histories of individual specimens impacts the MRI properties of their tissues, and thus the outcome of MRI, as we observed with DFL fishes. For instance, it is not uncommon for specimens to have been frozen and thawed prior to fixation, as this is often the most convenient method of preventing biological degradation in the field. However, freezing and thawing is a process that is known to degrade tissue at the cellular level, thus reduce its MR responses [28], [33]. In particular, freezing results in an irreversible loss of the water-holding capacity of tissue primarily due to protein denaturation and lipid oxidation, both of which have implications for T1- and T2-weighted imaging [33].

Although exposure to formalin fixatives does not significantly alter fish soft tissue anatomy as far as anatomical MRI is concerned [24], [25], [31], it has been shown to reduce MRI responses [37], diminishing SNR and hence image quality. MR properties can be restored to a large extent if these fixatives are removed [58]–[60]. However, we have found that the practice of storing specimens in alcohol, as is the tradition in fish collections, has a more significant and often irreversible impact on both the anatomy and MRI responses of tissues. Alcohols are known to reduce proton availability within tissues (e.g. through dehydration and extraction of lipids), therefore degrading MR responses and lowering tissue contrast [34]. Alcohol can also promote soft tissue shrinkage resulting in physical damage to specimens. For example, we frequently observed specimens where tissue shrinkage had caused muscles to pull away and detach from bones. As was demonstrated in Figure 2C, the presence of alcohol in specimen tissues also produces a significant residual MR signal intensity artefact which degrades T1-weighted images at 7T. Although not shown here, we experienced similar results at 3T when imaging fishes directly from alcohol. Waller et al. [25] also observed a similar effect at 4.7T. Although the removal of alcohol from specimen tissues may raise concerns from some collection managers, particularly with respect to delicate or rare specimens, we have found that removing specimens from storage jars and carefully rehydrating tissues to eliminate residual alcohol, greatly enhances the results of MRI. Furthermore, the MVC curators have not yet observed any detrimental effects to date in either the short or long term physical condition of imaged specimens. They have also been unable to detect any detrimental effects from the exposure of specimens to the gadolinium contrast agent or the perfluoropolyether media that we use.

In addition to the inherent differences in MRI responses of different tissues, chemical contrast agents offer a useful method of altering relaxation times to enhance these differences and increase tissue contrast [35]. Although primarily developed for administering intravenously in living specimens (e.g. manganese), some water-soluble contrast agents are sufficiently capable of passive diffusion into small excised tissue volumes within hours or days (e.g. gadolinium chelates and iodine) [36]–[38]. We have demonstrated here that, if given adequate time (e.g. 1–4 weeks), the contrast agent will diffuse evenly into the tissues of intact fishes and is thus also feasible for this application. We were therefore able to exploit the benefits of a gadolinium-based contrast agent in reducing T1 relaxation times, while maintaining a relatively long T2, to increase image SNR and also enhance the relative contrast of specific tissues and fluid compartments in our 7T specimens. This treatment also greatly helped to delineate anatomical structures during image segmentation. Overall, use of this contrast agent provided worthwhile improvements in the quality of our T1-weighted anatomical images, while reducing necessary signal averaging and therefore scan time (an important added benefit when paying for scanner time by the hour).

Securely mounting 7T specimens inside a pressurizable canister filled with perfluorinated fluid was effective for stabilizing unwanted movements and reducing the risk of specimen dehydration from exposure to air. This proved important for repeated and/or high field scans which may last for many hours at a time. Since they do not contribute any proton signal, perfluorinated fluids, such as Galden or Fomblin, act as a near ideal embedding media for T1-weighted high field MRI, providing specimens with a black, noise-free background in images. These fluids also minimize magnetic susceptibility effects at air-tissue surface boundaries, which otherwise cause local distortions in the magnetic field. They also help minimize the possibility of overloading of the coil and incidences of aliasing, or wrap-around, image artefacts, which may occur if specimens are immersed in water. Air can also be worked out of specimens thereby reducing the size or existence of unwanted air pockets and resulting susceptibilities. Therefore mounting specimens in this manner allows more consistent and stable imaging, particularly during long, high resolution scans. This was not so critical for 3T imaging since scan times were significantly shorter and scans were less susceptible to noise artefacts than higher field 7T scans.

The quality of MRI data also depends on the strength of the primary magnetic field of the scanner, as well as on the details of the imaging components, including the gradient coils whose strength determines the image resolution, and the RF (radio frequency) coils that have a significant impact on the SNR [20]. In order to optimize image quality and improve resolution, imaging coils should be well matched to the size and shape of the specimen. High field MRI systems typically provide better options, at least for specimens capable of fitting inside their hardware. Although the large bore of the 3T scanner facilitated a broader range of specimen sizes and faster scan times than the 7T scanner, the 7T provided much greater SNR and image resolution and as a result provided much greater anatomical detail. Therefore where possible, species capable of fitting inside our 7T hardware were preferentially imaged over larger species only suited to the 3T system. In either system, we found that with any given species, selecting the largest available specimen that would readily fit inside our best performing RF coil helped optimize SNR. However, body shapes and sizes of different fishes vary widely and are typically not optimal for imaging with most commercially available RF coil configurations (which are typically designed for human or small rodent body shapes). Although many of the DFL fishes were well matched for our circular or tubular-shaped 3T and 7T RF coils, e.g. bulky, cylindrical shaped fishes (such as sharks and tunas), and ball-shaped fishes (such as puffers and anglerfishes), most species had more compressed bodies (such as most perciforms and flatfishes) or thin, elongated bodies (such as eels). Although these fishes were not optimally shaped for our coils, this problem could be allayed to some degree by careful placement of specimens in the center of the coil and selecting an acquisition plane parallel to their thickest dimension.

Anatomical MRI ideally benefits from the combined acquisition of both T1-weighted and T2-weighted imaging. However, due to the large number of specimens being imaged for the DFL, it was necessary to economize on both scanning time and costs in order to realistically achieve our goals. Although T2-weighted imaging produces better overall image intensity contrast in tissue such as striated or smooth muscle in these museum fishes, T1-weighted imaging provides significantly better image intensity contrast between connective tissues (exhibiting longer T1), or fat and muscle tissues (exhibiting shorter T1) [28]. Our T1-weighted imaging protocol thus offered the best results for depicting the gross anatomy of major internal organs such as those of the alimentary tract, reproductive and excretory systems, etc, and provided sufficient image intensity and contrast for visualizing gross soft tissue anatomy in a diverse array of preserved fishes. T1 contrast can be manipulated by varying the excitation input power, or the flip angle, while allowing for short echo times (TE). It therefore has the advantage of also lending itself well to rapid 3D volume imaging while reducing distortions due to local susceptibility variations such as those caused by air pockets (commonly encountered in postmortem specimens). T1-weighted imaging also lends itself well to 3D isotropic imaging, which facilitates re-slicing of volume data sets along any orientation perpendicular to the acquisition direction without changing the in-plane resolution. The ability to re-slice data also minimizes complications when performing sophisticated 3D volumetric image analysis, such as image segmentation, where non-cubic voxels can result in directionally varying image distortions.

Despite an increase in access to MRI scanners over the past few years, the MRI properties of fresh and preserved fish tissues are not well characterized. Further research is therefore needed to better understand the causes, and mitigation, of inconsistencies in image quality that are frequently encountered when scanning museum specimens in particular. This can help determine the potential benefits of specific contrast agents and scanning protocols, both to enhance MRI results and reduce scan time and cost. Ideally, a standardized protocol for preserving fishes for MRI could be adopted by collection managers, with selected specimens made available for imaging prior to exposure to alcohol. For instance, immediately fixing freshly collected fishes in generous quantities of 10% neutral-buffered formalin, including the injection of liberal amounts of fixative into body cavities, would greatly improve specimen fixation and stabilize tissue pH and tonicity. This would allow specimens to remain in fixative long enough to ensure sufficient tissue fixation while minimizing the loss of mineralized tissues (which can be an issue when using unbuffered formalin). In addition, access to detailed records on the collection and preservation histories of specimens greatly assists in the selection of suitable candidates for MRI analysis.

### MRI and Fish Comparative Morphology

While non-invasive MR imaging and tissue segmentation methods have already been established as a tool for in situ characterization and quantification of fish soft tissues [22]–[33], [37], [61], the DFL is the first to demonstrate the feasibility of these methods for imaging a large and diverse array of preserved specimens for comparative morphology [8]–[14]. We have shown that large stores of fixed material from numerous public or private biological collections are now candidates for soft-tissue analysis with high-field MRI, thereby increasing their value as scientific resources. Although still heavily reliant on data from external body and hard tissue structures e.g. [62]–[64], descriptions of dissected soft tissue systems such as striated muscles [26], [65], internal organs, including gut, kidneys, venous system and reproductive structures [26], [66]–[69], have provided important insights into a number of fish phylogenies. High resolution MRI is slowly but increasingly contributing volumetric image data for comparative morphological studies of soft tissue systems in other taxa [7], [51], [57], and has enormous potential to contribute similar data in a broad range of fishes. For example, working in collaboration with the DFL, Chakrabarty et al. [14] has successfully applied high resolution MRI and segmentation methods to analyze the 3D morphology of the unique light organ systems (LOS) of ponyfishes, including a specimen holotype, acquired from a museum. This MRI dataset was used to reconstruct the character evolution of both internal and external features of LOS to investigate the evolution of sexual dimorphism in this soft tissue system. With the increasing availability of high quality MRI data, more sophisticated data analyses are possible. The most recent work of the DFL project has therefore been the complicated task of quantitative comparative morphology [12]–[14].

However, we recognize that using museum specimens for quantitative analyses of soft tissues raises some concerns, since their tissues typically exist in a range of qualitative states, as discussed above. Inconsistencies in preservation histories and exposure of specimens to alcohol often result in undesired changes to soft tissue morphology, primarily from tissue shrinkage and hardening, which may compromise their suitability for comparative morphology and quantitative analyses derived from segmentation. Though some of these problems can be mitigated with the careful selection of specimens, choosing tissues less susceptible to such postmortem changes, and with the use of specimen rehydration and MRI enhancing protocols, in general, museum specimens may not be as optimal as those that have been specifically collected and prepared for MRI. However, this does not necessarily detract from the value of digitizing collections and using specimens for comparative morphology [7], [8], [14], [51], [52], since soft tissue data collected with traditional dissection methods are similarly vulnerable to these problems. Sophisticated data analysis tools provide methods for computing a wide range of metrics with which to assess morphology and which can provide significant insight even in the presence of a certain amount of tissue shrinkage or distortion. However, the most obvious advantage that volumetric imaging (i.e. MRI) offers over dissection, is that it keeps the relative morphological architecture of the specimen intact, thereby eliminating problems of geometric distortion caused by dissection itself. Furthermore, high-resolution 3D imaging methods like MRI can allow a skilled morphologist to discern the subtle changes in specimen morphology that arise from tissue preservation, which may not be obvious from dissection. This paves the way for digital comparisons with fresh specimens or specimens specifically preserved for MRI. These types of analyses are of interest to the DFL and will be used to further refine the quantitative nature of the measurements that we have found so useful in our morphological studies that employ image segmentation (e.g. [8], [12]–[14]). However, for individual studies requiring high quality quantitative imaging of specific anatomy in certain species, it may be preferable to scan specimens whose soft tissues have been specifically preserved and prepared for MRI prior to being catalogued in a collection. Our digital library infrastructure incorporates any of these possibilities and thus offers users flexibility in addressing basic research questions and addressing issues relating to the creation, continuation, and dissemination of digital library materials.

There are even broader applications for MRI in morphological studies of fish. MRI is capable of measuring local water diffusion characteristics in tissue using a method called diffusion tensor imaging, which derives information about tissue micro-architecture from the variations in water diffusion magnitude and direction [18]. This can be used to determine details of muscle fiber orientations in cardiac tissue [70], or striated muscle [71], not otherwise visualized with conventional MR methods, and to reconstruct white matter fiber tracts in neural tissues [72], opening the possibility of mapping connections in fish neuroanatomy previously attainable only by painstaking reconstruction from histological sections e.g. [73]. Although these powerful 3D MR imaging technologies may never truly replace the resolution of traditional histology, or EM microscopy, improvements in temporal sampling and spatial resolution with each advancement in technology and image enhancement methods, means resolutions of less than 100 µm are becoming routine, with those less than 10 microns potentially viable [17]. MRI not only provides enormous possibilities for the quantitative assessment of fish anatomy with its numerous options of pulse sequences and pulse sequence parameters for optimizing image contrast [21] or measurement of quantities such as chemical species using MR spectroscopy (MRS: [74], [75], it also offers the possibility of quantitative assessment of in vivo physiology by measuring blood oxygenation and perfusion using functional MRI (fMRI) in living specimens [76].

While the current paper focuses on MRI, a variety of other 3D imaging modalities with potential applications for imaging zoological specimens have also recently become accessible. These include, X-ray computer tomography (CT and μCT) [77]–[80], optical projection tomography (OPT) [81], synchrotron-radiation X-ray tomography (SRXTM) [82], [83], and ultrasonography [84], each with their own strengths and weaknesses [85]. Comparative morphology can be even more greatly served by exploiting the various strengths that many of these digital imaging technologies offer, using a complementary multimodal imaging approach [16], [23], [86]. The choice of modality may be determined by any of the following considerations: the size and shape of the specimen; how it is preserved; the composition of tissues of interest; level of desired resolving power; the speed at which data can be acquired; requirements for volume reconstruction; and expense. Although our current focus remains MRI, data acquired from any, or all, of these imaging methods can be accommodated into the current infrastructure of the DFL.

Both CT and MRI produce 3D volumetric image data that are composed of individual volume elements (voxels) over which the local signal is averaged, an important distinction from imaging methods (e.g., X-ray and light photography) that simply produce 2D picture elements (pixels). While voxel data are frequently viewed as 2D slices of pixels, it is important to understand that the information in a voxel is an averaged physical quantity that depends upon the particular acquisition method and the subsequent influence of the tissue on the signal. MRI, for example, can acquire data with a wide range of sensitivities, such as the amount of water, water diffusion, water perfusion, etc. The voxel data represent localized averages of whatever quantity the acquisition is sensitized to, and the display data are then some representation (e.g., the magnitude) of this quantity. Because these data are collected in volumes, they can be displayed in a variety of ways, such as in slices, which can be used to create 2D images or movies, or as volumetric or surface renderings, which allow the creation of interactive 3D graphical models of anatomy. Displayed slices need not be chosen to conform to those collected but can be generated by re-slicing the data along any orientation. These aspects of truly 3D volumetric data make these imaging techniques powerful tools not just for data storage, but also for the analysis and interpretation of specimen morphology.

Recent developments in software methods have enabled the creation of a range of single-species digital atlases and databases based on 3D volumetric image data [5], [51], [87]–[99] and large-scale voxel-based imaging libraries, such as The Digital Morphology Library [100] and the DFL presented here [6]. However, with the enormous volumes of quantitative data that 3D imaging technologies inevitably generate, there is a critical need for computational tools that provide users with the ability for efficient data mining and analysis of these very large image datasets [101]–[103]. Enormous potential will then emerge to rapidly, and objectively, digitize, quantify, and analyze vast arrays of anatomical and morphological features, in a vast and extremely diverse range of species. The goal of the DFL is therefore to develop the critical technological methods for scanning, visualizing, analyzing, archiving and disseminating high quality zoological digital imaging data, primarily focused on specimens housed in museum collections. How the data and methods are subsequently used is entirely up to the “user”. To date, our methods and data have been successfully applied to a broad range of research and educational projects [8]–[15], [104]. We do recognize that there is a lot more work to be done in optimizing web-based tools for both research and educational purposes. These are ongoing in our lab. However, the critical infrastructure necessary to incorporate these future modifications has been established.

### Conclusions and Future Work

With the DFL database and website, we are able to efficiently present complex 3D digital data for a vast array of imaged specimens over the internet, thereby increasing the accessibility of anatomical data derived from museum specimens to a very broad audience. Three-dimensional volumetric digital imaging offers great promise for zoological collections and their use in comparative anatomy and morphology. It facilitates permanent archiving of digital data that can then be easily disseminated over the Internet. Such data can also be explored with a wide variety of sophisticated computation methods for morphological analysis and visualization, and re-analyzed when newer methods are developed. But these benefits come at the cost of increasingly sophisticated (and expensive) imaging devices that often require specialized facilities and staff, meticulous specimen preparation methods designed to enhance tissue contrast while mitigating imaging artefacts, exceedingly complex volumetric data that require advanced computational methods for analysis and visualization, and sophisticated data archival schemes that facilitate efficient data storage, search, and retrieval. At present, the DFL project utilizes a wide range of sophisticated visualization and analysis methods in collaboration with its users. However, implementing these for efficient on-line usage is significantly more complicated than their implementation on the users' host computer. Improvements on porting these tools to web-based tools will be an ongoing endeavor. We emphasize, however, that we have developed a computational infrastructure in which visualization and analysis are directly and automatically linked to the database and accessible via the web. This infrastructure will facilitate future improvements to the web based tools. Importantly, we view archival, dissemination, visualization and computational issues as inter-related and thus have designed and developed their implementation in concert. In this paper we have described our effort to accomplish this within the context of a single imaging modality (MRI) for a specific group of organisms (fishes) from a single scientific collection (MVC). While the data acquisition details are specific to MRI, they are not particularly specific to fish. And the archiving, analysis and visualization are generally applicable to other imaging modalities. In conclusion, our Digital Fish Library provides a model for the development of a digital library of volumetric imaging data for quantitative comparative morphology.
